# Aryl hydrocarbon receptor antagonism before reperfusion attenuates cerebral ischaemia/reperfusion injury in rats

**DOI:** 10.1038/s41598-020-72023-5

**Published:** 2020-09-10

**Authors:** Jae-Im Kwon, Hwon Heo, Su Jeong Ham, Yeon Ji Chae, Do-Wan Lee, Sang Tae Kim, Joongkee Min, Yu Sub Sung, Kyung Won Kim, Yoonseok Choi, Dong Cheol Woo, Chul-Woong Woo

**Affiliations:** 1 Asan Institute for Life Sciences, Asan Medical Center, 88, Olympic-ro 43-gil, Songpa-gu, Seoul, 05505 Republic of Korea; 2 Convergence Medicine Research Center, Asan Medical Center, 88, Olympic-ro 43-gil, Songpa-gu, Seoul, 05505 Republic of Korea; 3 Clinical Research Center, Asan Medical Center, 88, Olympic-ro 43-gil, Songpa-gu, Seoul, 05505 Republic of Korea; 4 Department of Radiology, Asan Medical Center, 88, Olympic-ro 43-gil, Songpa-gu, Seoul, 05505 Republic of Korea; 5 Medical Research Institute, Gangneung Asan Hospital, 38, Bangdong-gil, Sacheon-myeon, Gangneung-si, Gangwon-do Republic of Korea

**Keywords:** Neurological disorders, Diseases of the nervous system

## Abstract

Aryl hydrocarbon receptor (AhR) antagonism can mitigate cellular damage associated with cerebral ischaemia and reperfusion (I/R) injury. This study investigated the neuroprotective effects of AhR antagonist administration before reperfusion in a rat stroke model and influence of the timing of AhR antagonist administration on its neuroprotective effects. Magnetic resonance imaging (MRI) was performed at baseline, immediately after, and 3, 8, and 24 h after ischaemia in the sham, control (I/R injury), TMF10 (trimethoxyflavone [TMF] administered 10 min post-ischaemia), and TMF50 (TMF administered 50 min post-ischaemia) groups. The TMF treatment groups had significantly fewer infarcts than the control group. At 24 h, the relative apparent diffusion coefficient values of the ischaemic core and peri-infarct region were significantly higher and relative T2 values were significantly lower in the TMF10 groups than in the control group. The TMF treatment groups showed significantly fewer terminal deoxynucleotidyl transferase dUTP nick-end labelling positive (+) cells (%) in the peri-infarct region than the control group. This study demonstrated that TMF treatment 10 or 50 min after ischaemia alleviated brain damage. Furthermore, the timing of AhR antagonist administration affected the inhibition of cellular or vasogenic oedema formation caused by a transient ischaemic stroke.

## Introduction

In acute stroke patients, restoring blood supply as soon as possible is considered the optimal treatment to preserve neurological function. The re-establishment of blood supply can be achieved via intravenous administration of a thrombolytic agent, such as recombinant tissue plasminogen activator, or by mechanical removal of the thrombus^1^. However, after effective thrombolytic therapy for stroke, cerebral ischaemia and reperfusion (I/R) injury can occur and cause more severe secondary damage to brain tissue^2^. The pathophysiological mechanisms of cerebral I/R injury are very complex and include oxidative stress, glutamate/neurotoxin release, inflammation, and apoptosis^3–6^. Therefore, many research groups have attempted to develop practical and effective strategies for preventing I/R injury. However, little is known regarding the therapeutic strategies to alleviate I/R injuries in clinical practice.

**Figure 1:**
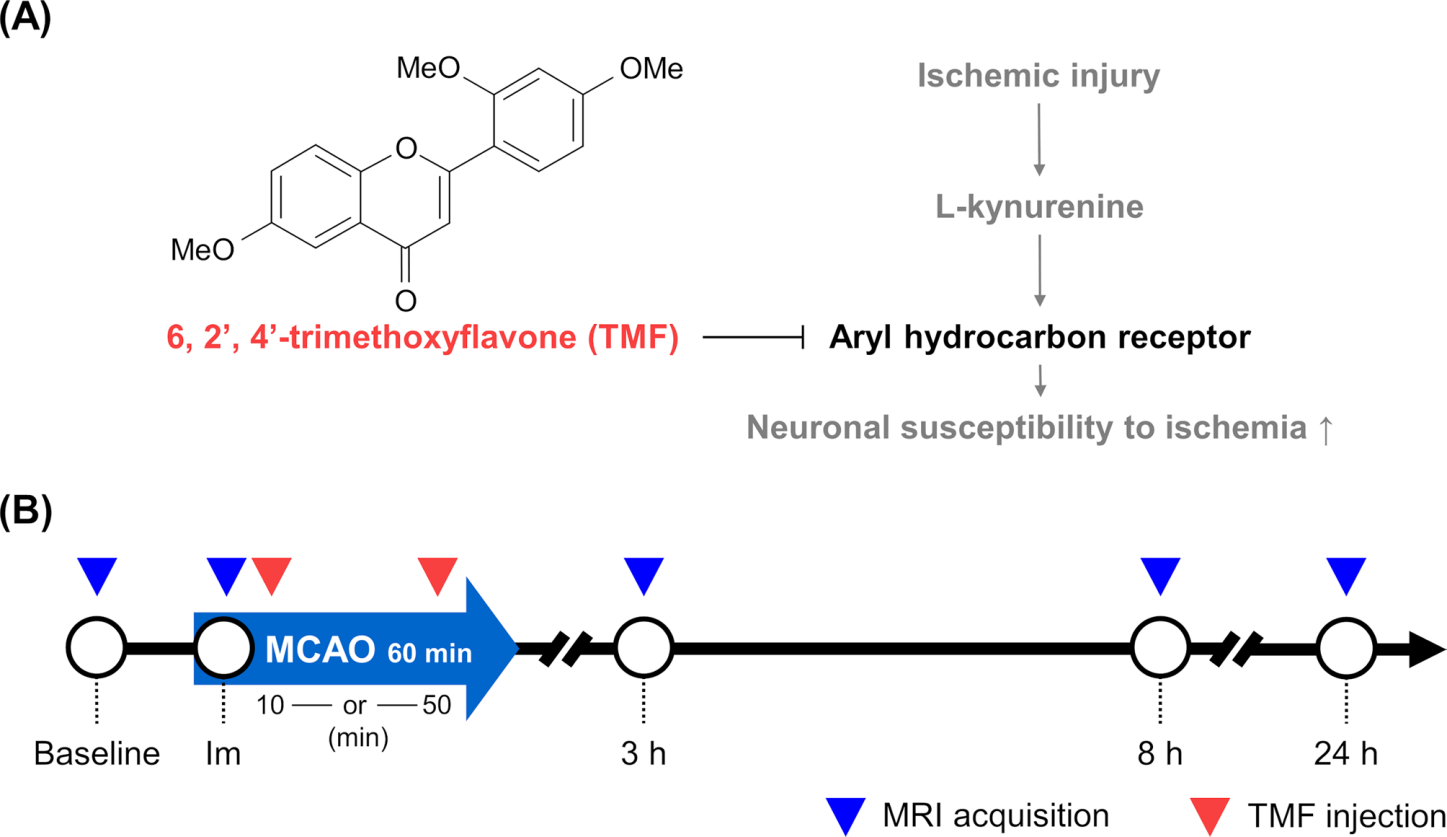
Inhibition of Aryl hydrocarbon receptor (AhR) activation and magnetic resonance imaging (MRI) acquisition (**A**) Schematic of trimethoxyflavone (TMF) treatment for AhR antagonism. (**B**) Protocol of AhR treatment and MRI acquisition during the follow-up period.

The aryl hydrocarbon acceptor (AhR) is a basic helix-loop-helix/Per-Arnt-Sim transcription factor that can be activated by various endogenous and exogenous ligands^7,8^. Upon ligand binding, the AhR translocates from the cytoplasm to the nucleus and heterodimerises with an aryl hydrocarbon nuclear translocator (ARNT) to bind DNA and alter gene expression^9^. A recent study suggested that cerebral ischaemia leads to the activation of AhR and aggravates neuronal damage^10^. Pharmacological manipulation of AhR activation has also been shown to modulate neuronal damage due to transient cerebral artery occlusion in vivo (Fig. 1A)^10,11^; however, although it was demonstrated that early inhibition of AhR conferred neuroprotection in ischaemic stroke, whether inhibition was effective at later time points has not been revealed. Furthermore, there have been no reports regarding the optimal timing of inhibition of AhR, which is an important consideration in the context of ischaemic stroke.

In this study, we investigated the neuroprotective effects of AhR antagonism before reperfusion in a rat model of stroke induced by transient middle cerebral artery occlusion (tMCAO) and reperfusion. Furthermore, we used in vivo magnetic resonance imaging (MRI) and histochemical analyses to assess whether the timing of AhR antagonist administration influences its neuroprotective effects.

## Results

### Animal modelling

**Figure 2:**
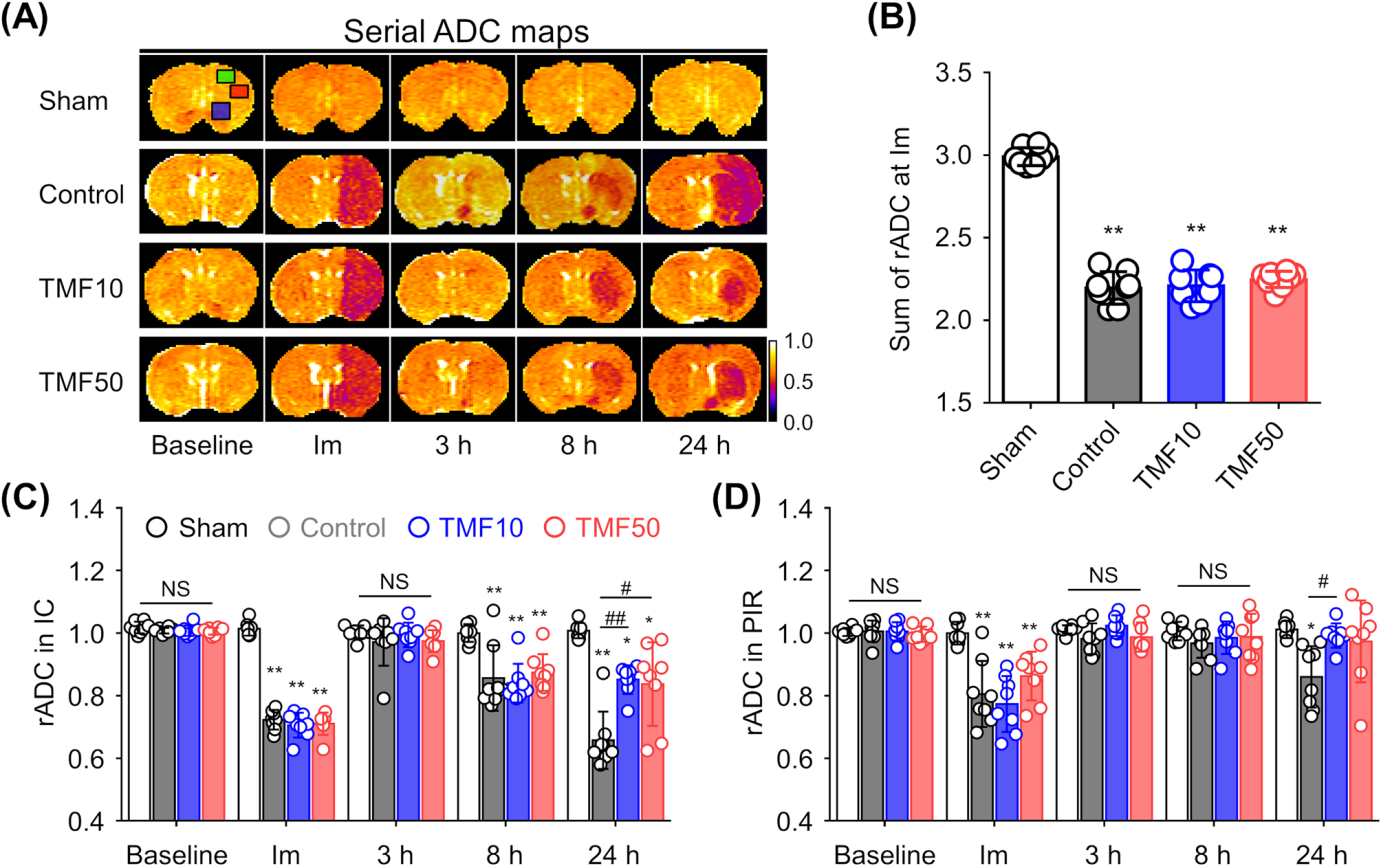
Effect of TMF treatment on apparent diffusion coefficient (ADC) values (**A**) Mean ADC values were measured in the ipsilateral ischaemic core (IC, red square), peri-infarct region (PIR, green square), anterior choroidal and hypothalamic region (AHR, blue square), and contralateral regions on ADC maps during the follow-up period. (**B**) Combined relative ADC (rADC) values immediately after ischaemia were decreased in groups with induced ischaemia. (**C**,**D**) The rADC values of the (**C**) IC and (**D**) PIR were plotted against time. Data are represented as means ± SDs (n = 8 rats in each group). *P < 0.05 vs. sham group; **P < 0.001 vs. sham group; ^#^P < 0.05 vs. control group; ^##^P < 0.001 vs. control group.

The success rate of tMCAO modelling was 70.5% (24 animals were included, with 34 undergoing surgery). The success rate for each group and causes for exclusion from the study are presented in Supplementary Table S1 online. All included rats in the TMF10, TMF50, and control groups exhibited marked decreases in the regional cerebral blood flow (rCBF) after ischaemia (i.e., > 70% reduction) compared to baseline rCBF in the sham-operated group (Supplementary Table S2 online). Furthermore, immediately after ischaemia, all included rats in the TMF10 [trimethoxyflavone (TMF) administered 10 min post-ischaemia], TMF50 (TMF administered 50 min post-ischaemia), and control groups showed significant decreases in the sum of relative apparent diffusion coefficient (rADC) values (i.e., > 20% reduction) compared to the sum of rADC values in the sham-operated group (P < 0.001, Fig. 2A,B). Rats in the sham-operated group did not show any reduction in the rCBF and sum of rADC values. There was no significant difference in rCBF reduction (P > 0.05) and the sum of rADC values among the TMF10, TMF50, and control groups (P > 0.05).

### Effect of TMF treatment on ADC and T2 values

Figure 2A shows the evolution of lesions from apparent diffusion coefficient (ADC) maps at baseline, immediately after, and 3, 8, and 24 h after ischaemia in the representative rats from each group. Ischaemic/infarcted areas in the ipsilateral hemisphere showed lower ADC values than those in the contralateral hemisphere, thus reflecting the degree of cellular oedema. In the sham-operated group, the rADC values showed little change over time (Fig. 2C,D). In the control, TMF10, and TMF50 groups, the rADC values in the ischaemic core (IC) and peri-infarct region (PIR) decreased immediately after ischaemia, but they almost recovered to the baseline level at 3 h. At 8 h after ischaemia, the rADC values of the IC and PIR decreased in the control, TMF10, and TMF50 groups. The IC (P < 0.001) and PIR (P < 0.05) of the TMF10 group had significantly higher rADC values than those of the control group at 24 h. Although the rADC values of the IC (P < 0.05) and PIR (P = 0.058) were higher in the TMF50 group than in the control group at 24 h, the difference was statistically significant only for the rADC values of the IC.

**Figure 3:**
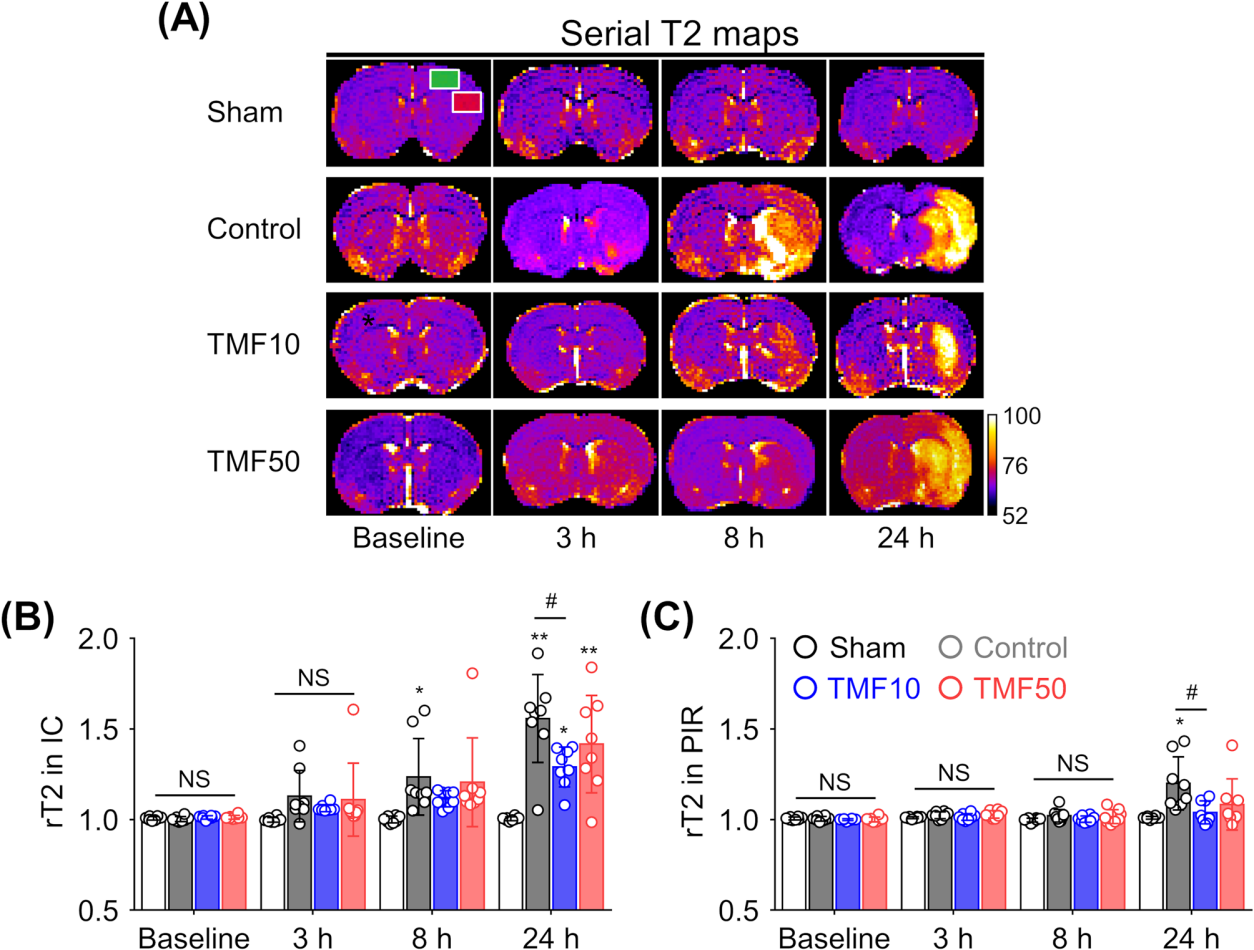
Effect of TMF treatment on T2 values (**A**) Mean T2 values were measured in the ipsilateral IC (red square), PIR (green square), and contralateral regions on T2 maps during the follow-up period. (**B**,**C**) The relative T2 (rT2) values of the IC (**B**) and PIR (**C**) were plotted against time. Data are represented as means ± SDs (n = 8 rats in each group). *P < 0.05 vs. sham group; **P < 0.001 vs. sham group; ^#^P < 0.05 vs. control group.

Figure 3A shows the evolution of lesions from T2 maps at baseline and at 3, 8, and 24 h after ischaemia in the representative rats from each group. The ischaemic/infarcted areas in the ipsilateral hemisphere showed higher T2 values than those in the contralateral hemisphere, thus reflecting the degree of vasogenic cerebral oedema. In the sham-operated group, the T2 values did not differ over time (Fig. 3B,C). On serial T2 maps, the relative T2 (rT2) of the IC increased over time in the control, TMF10, and TMF50 groups, but the rT2 of the PIR remained almost unchanged until 8 h after ischaemia in the three groups and increased at 24 h. The rT2 values of the IC (P < 0.05) and PIR (P < 0.05) were significantly lower in the TMF10 group than in the control group at 24 h after ischaemia. The rT2 values of the IC (P = 0.456) and PIR (P = 0.153) were lower in the TMF50 group than in the control group at 24 h after ischaemia; however, no statistically significant difference was observed.

### Reduction in infarct volume and neuronal loss in TMF-treated animals

**Figure 4:**
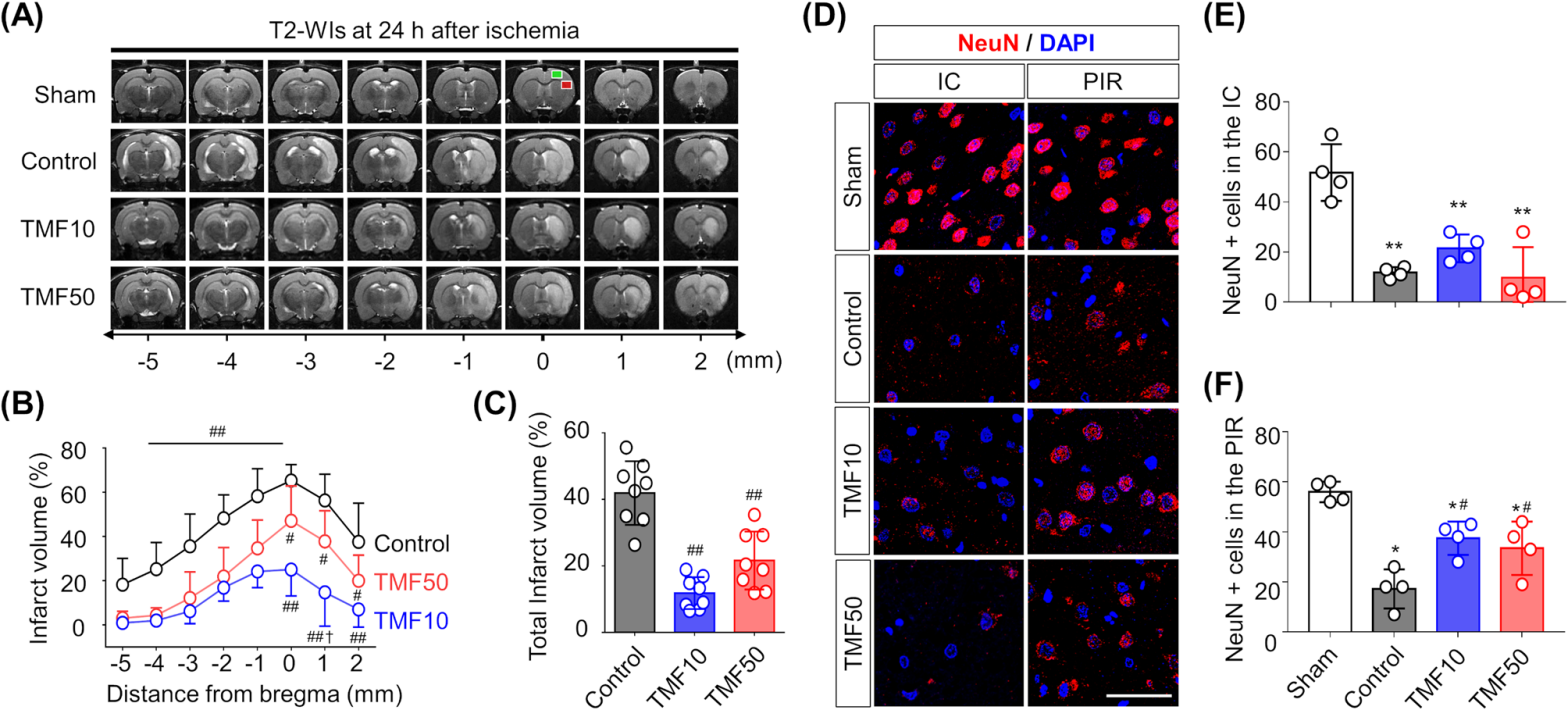
Infarct volumes and neuronal nuclear (NeuN) positive (+) cells (**A**) Infarct volumes at 24 h after ischaemia were measured in the nearest eight slices of T2-weighted imaging (T2-WI) on stereotactically predetermined coronal planes. (**B**) The infarct volumes in all slices were the lowest in the TMF10 group, followed by the TMF50 and control groups. (**C**) Total infarct volumes were significantly lower in the TMF treatment groups than in the control group. Data are represented as means ± SDs (n = 8 rats in each group). (**D**) Representative NeuN staining (magnification × 630, scale bar = 20) from the IC (red square) and PIR (green square) in (**A**). (**E**,**F**) Quantification of the effect of TMF treatment on NeuN + cells in the (**E**) IC and (**F**) PIR. Data are represented as means ± SDs (n = 4 rats in each group). *P < 0.05 vs. sham group; **P < 0.001 vs. sham group; ^#^P < 0.05 vs. control group; ^##^P < 0.001 vs. control group; ^**†**^P < 0.05 vs. TMF50 group.

Figure 4A shows infarct lesions on T2-weighted imaging (T2-WI) scans at 24 h after ischaemia in the representative rats from each group. The infarct volume in the eight slices of T2-WI and total infarct volumes were the lowest in the TMF10 group, followed by in the TMF50 and control groups, which is indicative of the neuroprotective effect of TMF. The infarct volume in each of the eight slices of T2-WI showed that the TMF10 group had significantly lower values than the control group (P < 0.001, Fig. 4B). The TMF50 group also had significantly lower values than the control group (distance from bregma: − 5 to − 1, P < 0.001; distance from bregma: 0 to 2, P < 0.05). The infarct volume at a 1-mm distance from bregma in the TMF10 group was significantly lower than that in the TMF50 group (P < 0.05). In the total infarct volume analysis, both TMF treatment groups showed significantly lower volumes than the control group (TMF10 vs. control, P < 0.001; TMF50 vs. control, P < 0.001), but no significant difference was observed between the TMF10 and TMF50 groups (Fig. 4C).

The number of neuronal nuclear (NeuN) positive (+) cells was counted in the sham, control, TMF10, and TMF50 groups (Fig. 4D). In the IC, the number of NeuN + cells was significantly decreased in the control, TMF10 and TMF50 groups compared to that in the sham-operated group (P < 0.001, Fig. 4E). There was no significant difference in the number of NeuN + cells among the TMF10, TMF50, and control groups (P > 0.05). In the PIR, the number of NeuN + cells was significantly decreased in the control, TMF10 and TMF50 groups compared to that in the sham-operated group (P < 0.05, Fig. 4F). Both TMF treatment groups showed a significantly lower number of NeuN + cells than the control group (TMF10 vs. control, P < 0.05; TMF50 vs. control, P < 0.05), but there was no significant difference between the TMF10 and TMF50 groups (P = 0.880).

### Suppression of apoptosis with TMF treatment

**Figure 5:**
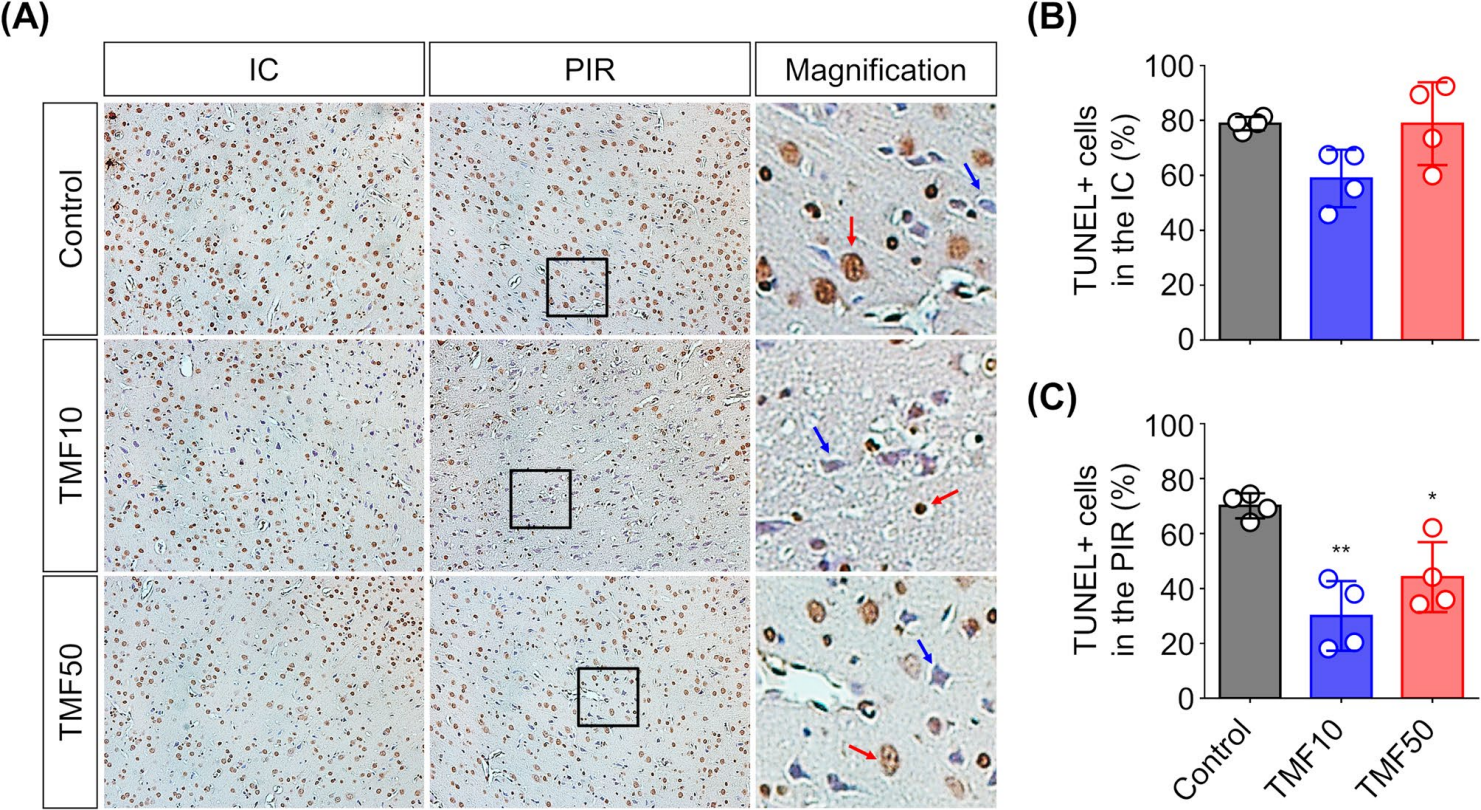
Reduction in apoptosis after TMF treatment (**A**) Representative photomicrographs of terminal deoxynucleotidyl transferase dUTP nick-end labelling (TUNEL) staining in the IC and PIR (magnification × 200, scale bar = 100). The right row shows enlarged versions of the black boxes in the middle row (scale bar = 20 μm). Red arrows indicate apoptotic cells; blue arrows indicate viable cells. (**B**,**C**) Quantification of the effect of TMF treatment on TUNEL positive (+) cells (%) in the (**B**) IC and (**C**) PIR. Data are represented as means ± SDs (n = 4 rats in each group). *P < 0.05 vs. control group; **P < 0.001 vs. control group.

To quantify the terminal deoxynucleotidyl transferase dUTP nick-end labelling (TUNEL) positive (+) cells (%) in the control, TMF10, and TMF50 groups, TUNEL staining was used, and TUNEL + cells (round brown nuclei) were observed in all groups (Fig. 5A). In the IC, the rate of TUNEL + cells (%) did not differ significantly between the TMF-treated and control groups (TMF10 vs. control, P = 0.063; TMF50 vs. control, P = 1.000; Fig. 5B). In the PIR, the rate of TUNEL + cells (%) was the lowest in the TMF10 group, followed by the TMF50 and control groups (Fig. 5C). Both TMF treatment groups showed significantly lower rates of TUNEL + cells (%) than the control group (TMF10 vs. control, P < 0.01; TMF50 vs. control, P < 0.05), but there was no significant difference between the TMF10 and TMF50 groups (P = 0.204).

### Inhibition of AhR nuclear translocation by TMF administration

**Figure 6:**
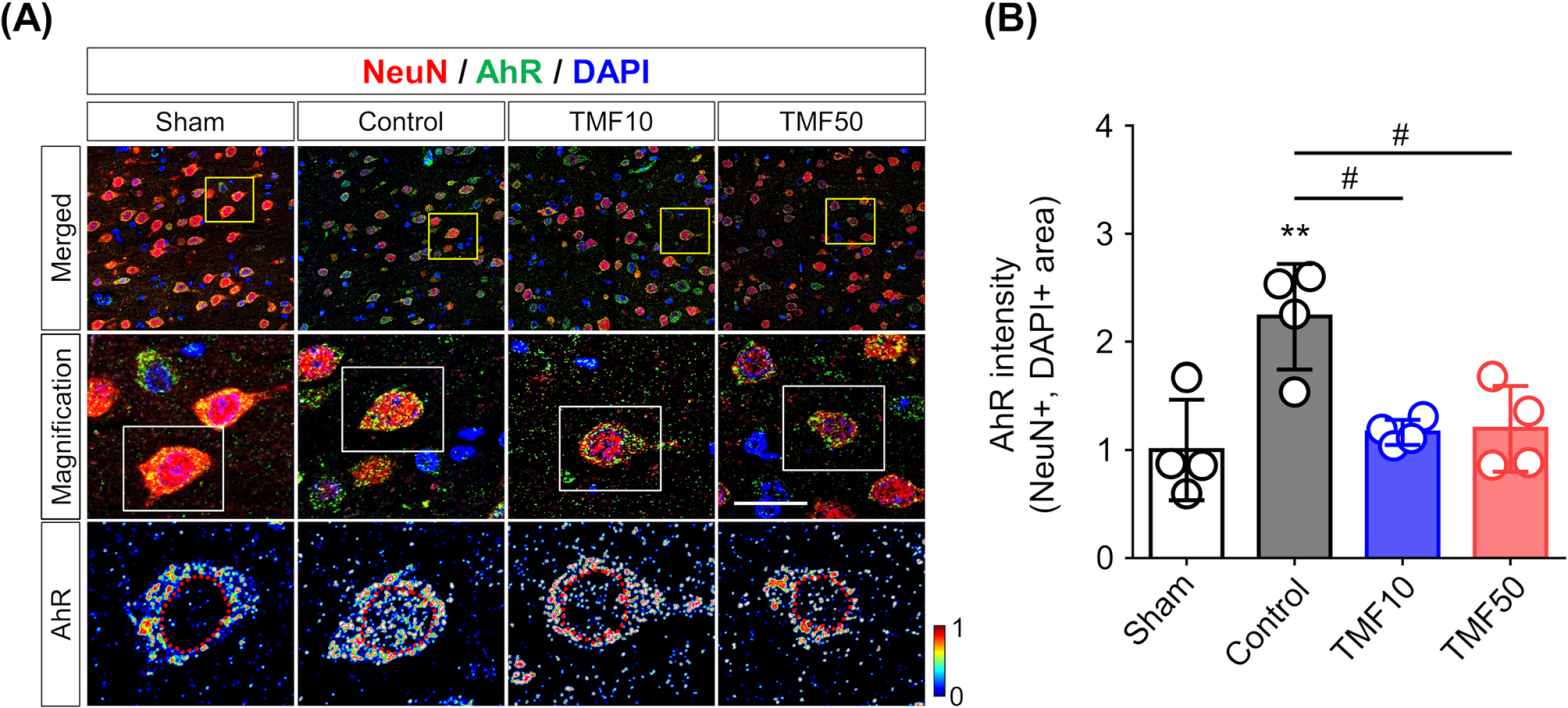
Inhibition of AhR activation by TMF treatment (**A**) Representative merged images of AhR, NeuN, and 4′,6-diamidino-2-phenylindole (DAPI) staining of the PIR 24 h after ischaemia (upper row, magnification × 630). Yellow boxes indicate the regions displayed at higher magnification from the middle row (scale bar = 20 μm). The bottom row shows photomicrographs of AhR signal intensity, which are enlarged versions of the white boxes in the middle row. The red dotted lines outline the nucleus. (**B**) The signal intensity of nuclear AhR in the control group was significantly higher than that in the sham and TMF treatment groups. **P < 0.01 vs. sham group; ^#^P < 0.05 vs. control group.

Immunofluorescence staining was performed to observe changes in the AhR location due to cerebral I/R and AhR antagonist administration. Figure 6A shows the locational change in AhR immunoreactivity at 24 h after ischaemia in the PIR. The signal intensity of nuclear AhR in the control group was significantly higher than that in the sham-operated (P < 0.01) and TMF treatment groups (P < 0.05), but there was no significant difference between the TMF10 and TMF50 groups (P = 0.999, Fig. 6B).

## Discussion

In the present study, we evaluated the neuroprotective effects of AhR antagonism before reperfusion and assessed the relationship between the timing of AhR antagonist administration and subsequent protective effects against cerebral I/R injury in rats. TMF-treated rats showed lower infarct volumes, number of NeuN + cells, and apoptosis levels than untreated rats at 24 h after ischaemia. However, there were no significant differences in the infarct volume, number of NeuN + cells, or apoptosis levels between TMF-treated rats at 10 and 50 min after ischaemia. Consistent with these findings of the infarct volume, number of NeuN + cells, and apoptosis levels, rats treated with TMF at 10 min after ischaemia showed a significant decrease in the formation of cellular oedema (rADC) and vasogenic oedema (rT2) in the IC and PIR at 24 h after ischaemia^12,13^. In contrast, only rats treated with TMF 50 min after ischaemia showed a significant decrease in the formation of cellular oedema in the IC. These results suggest that the inhibition of AhR activation before reperfusion alleviates brain damage due to apoptosis. Furthermore, the timing of TMF treatment after ischaemia affects the inhibition of cellular and vasogenic oedema formation due to cerebral I/R.

AhR remains inactive in the cytoplasm under normal conditions; when activated by ligands, it is translocated to the nucleus and heterodimerises with ARNT to bind DNA and to alter gene expression^9^. Recently, Cuartero et al. suggested that AhR is activated by in vivo experimental strokes caused by middle cerebral artery occlusion (MCAO)^10^. It was also reported that the administration of AhR antagonists at 10 min after ischaemia was beneficial when ischaemia was followed by recanalization^10^. However, they did not investigate whether the administration of AhR antagonists is effective at later time points after ischaemia. In this study, the infarct volume, number of NeuN + cells, and apoptosis level significantly decreased in rats treated with TMF at 50 min as well as at 10 min after ischaemia. These results indicate that AhR antagonism at a delayed time point after ischaemia is also effective in suppressing cerebral I/R injury.

Recently, Shi et al. reported that compared to that in the pre-MCAO condition, regional cerebral blood flow rapidly decreased to about 25% in the ischaemic region immediately after MCAO^14^. This means that even during MCAO, a small amount of AhR antagonist can be supplied to the ischaemic region through the blood. Recent studies have also suggested that AhR is activated by MCAO, resulting in a decrease in cAMP response element-binding protein-dependent signalling, which exacerbates brain damage^10,11^. These reports indicate that there may be a difference in ischaemic damage depending on the timing of AhR antagonist administration before reperfusion. Furthermore, differences in pharmacokinetic properties caused by gaps in the timing of administration of AhR antagonists after ischaemia can lead to differences in the efficiency of AhR activation inhibition during reperfusion, which can induce differences in cerebral I/R injury. However, in this study, the timing of AhR antagonist administration after ischaemia did not lead to differences in cerebral I/R injury. These results were obtained using a TMF concentration of 5 mg/kg, as suggested by Cuartero et al., but the appropriate dosage range should be further investigated, as corresponding differences in therapeutic effects may be observed^10^.

The timing of administration of AhR antagonists after ischaemia affected the inhibition of the formation of cellular and vasogenic oedemas due to cerebral I/R. Cellular oedema is known to be characterised by abnormal intracellular accumulation of water in brain cells, resulting in cellular swelling^15^. Vasogenic oedema results from the disruption of the blood–brain barrier and allows intravascular proteins and fluid to penetrate the interstitial space of the brain parenchyma^16^. These excess accumulations of fluid in the intracellular or extracellular spaces lead to an increase in the brain volume and intracranial pressure, causing an irreversible impairment of nerve function^17^. In this study, we used MRI to indirectly confirm that AhR antagonists reduce brain oedema formation and that the timing of AhR antagonist administration after ischaemia is an important factor influencing brain oedema formation. However, because the mechanism by which AhR is involved in the formation of brain oedema caused by cerebral I/R has not been elucidated, further research is needed to clarify the underlying mechanism.

There are two limitations in this study. First, we only observed the inhibitory effect of cerebral I/R injury in animals administrated with AhR antagonists before reperfusion. In clinical practice, however, patients with acute ischaemic stroke prioritise recovery of blood supply using standard clinical treatment strategies such as intravenous thrombolysis and intra-arterial thrombectomy^18^. Therefore, it is considered to be clinically more valuable to evaluate changes in cerebral I/R injury when AhR antagonists are administered after reperfusion, and further studies are needed in the future. Second, we showed that the signal intensity of nuclear AhR at 24 h after ischaemia was significantly higher in animals with cerebral I/R injury than in the sham-operated and TMF treatment animals. This means that the translocation of AhR to the nucleus can be one of the factors that play an important role in the progression of cerebral I/R injury. However, this study observed changes in AhR only at 24 h after ischaemia and did not reveal how the AhR nuclear signal changes after ischaemia or how long it persists. Cuartero et al. reported that the nuclear/cytoplasmic ratio of AhR increased by 2- and 2.4-fold in the brains of animals exposed to MCAO, respectively, compared to the sham animals at 1 and 18 h after ischaemia, returning to basal levels after 7 days^10^. Thus, although this study showed that the AhR signal intensity of the nucleus increased only 24 h after ischaemia, it is assumed that it was likely to have increased around 24 h.

Our findings demonstrate that the administration of AhR antagonists before reperfusion has neuroprotective effects and that the timing of AhR antagonist administration affects its neuroprotective effect on cerebral I/R injury. We suggest that appropriate AhR antagonist activity is a potential therapeutic approach for cerebral I/R injury. Nevertheless, to assess the potential clinical applications of AhR antagonist administration, further studies are needed to elucidate the exact time-dependency of the efficacy of AhR antagonists against cerebral I/R injury and to elucidate the underlying mechanisms of the observed neuroprotective effects.

## Methods

### tMCAO model

Male Sprague–Dawley rats were used in the experiments (8 weeks old; weight, 290–310 g; Orient Bio, Pyeongtaek, Republic of Korea). All rats were individually housed in standard plastic cages and maintained on a 12-h light–dark cycle (lights on at 08:00 A.M.) at an ambient temperature of 24.0–25.0 °C with free access to food and water.

Focal cerebral I/R injury was induced in the tMCAO rat model. The rats were initially anaesthetised using 5% isoflurane in 70% N_2_O/30% O_2_ (flow rate, 1.0 L/min), and anaesthesia was maintained using 2% isoflurane during surgery. To induce MCAO, an 18–20 mm commercial silicon rubber–coated 5–0 nylon monofilament (tip diameter, 0.35–0.37 mm) was advanced from the external carotid artery into the lumen of the internal carotid artery to block the origin of the middle cerebral artery. After 60 min, the inserted monofilament was gently removed to induce reperfusion. Sham-operated rats were manipulated in the same manner without inducing MCAO and reperfusion.

To identify rats that have successfully established MCAO, a laser Doppler flow (LDF) monitoring device (VMS-LDF, Moor Instruments, Devon, UK) was used to monitor the rCBF before and after MCAO^19^. For the placement of the LDF probe, a burr hole (diameter, 2 mm) was drilled 2 mm posterior and 6 mm laterally to the bregma and care was taken not to damage the underlying dura mater^20^. Rats that showed no significant rCBF reduction after MCAO (at least 70% decrease from the baseline value) were excluded from the experimental group^21^. Sham-operated rats were manipulated in the same manner without MCAO.

### Drug administration

Five mg/kg of 6,2′,4′-trimethoxyflavone (TMF; Sigma-Aldrich, St. Louis, MO) dissolved in dimethyl sulfoxide (DMSO; Sigma-Aldrich) was used as an AhR antagonist. The same volume of DMSO was used for the vehicle-treated control group. TMF and the vehicle were administrated via intraperitoneal injection.

### Experimental groups

The sample size for fixed-effect, omnibus, and one-way analysis of variance (ANOVA) were calculated using G-Power 3.1 software. Based on an 80% power and alpha level set at 5%, four groups (32 animals, n = 8/group) were needed^22,23^. Rats were randomly divided into the (1) sham-operated group with no I/R modelling or vehicle injection (n = 8), (2) control group with I/R modelling and vehicle injection (n = 8), (3) TMF10 group with I/R modelling and injection of the drug at 10 min after ischaemia (n = 8), and (4) TMF50 group with I/R modelling and injection of the drug at 50 min after ischaemia (n = 8). If death occurred during the follow-up period, additional rats were included to meet the sample size requirement.

### MRI

MRI was performed using a 7.0 T Bruker PharmaScan 70/16 MRI system (Bruker BioSpin, Ettlingen, Germany) with the ParaVision 6.0.1 software in the configuration involving a 72-mm transmit volume coil and a rat brain surface receiver coil. All animals were anaesthetised through a mask via spontaneous inhalation of 2% isoflurane in 70% N_2_O/30% O_2_ (flow rate, 1.0 L/min). Respiration was monitored, and the temperature of the rats was steadily maintained at 37.5 ± 0.5 °C using a circulating water bath system (CW-05G Heated Circulating Water Bath; MIDSCI, St. Louis, USA).

MRI data were obtained at baseline, immediately after, and 3, 8, and 24 h after ischaemia (Fig. 1B). The MRI protocol involved T2-WI, T2 mapping, and spin-echo echo-planar diffusion-weighted imaging. The parameters for each sequence are shown in Supplementary Table S3 online. Quantitative T2 maps and ADC maps were created using ParaVision’s built-in post-processing tools (FitInISA macro). T2 maps were not obtained immediately after ischaemia because T2 mapping is insensitive to ischaemic alteration.

### MRI analysis

All MRI data were analysed using the ImageJ software (National Institutes of Health, Bethesda, Maryland; https://rsbweb.nih.gov/ij/) by an observer who was blinded to grouping information.

The ADC values were measured in the IC, PIR, and anterior choroidal and hypothalamic region (AHR) of the ipsilateral and contralateral hemispheres to identify animals in which tMCAO was successfully induced; then, the rADC value was calculated as the ratio of the ipsilateral value to contralateral value (Fig. 2). Recently, it has been reported that the sum of rADC values in the IC, PIR, and AHR in the acute phase can predict permanent cerebral I/R injury in rats^24^.

The rADC and rT2 values, indicating the formation of cellular and vasogenic oedemas, respectively, were measured in the IC and PIR during the follow-up period (Figs. 2A, 3A)^12,13^. The rT2 value was calculated in the same way as the rADC value.

The infarct volume at 24 h after ischaemia was measured in the nearest eight slices of T2-WI on stereotactically predetermined coronal planes (from posterior − 5.0 to anterior + 2.0 mm, relative to bregma) and was calculated as follows: percentage of infarct volume = (contralateral hemisphere volume − ipsilateral intact volume)/contralateral hemisphere volume × 100%. The total infarct volume (%) was the sum of the infarct volumes of each slice (Fig. 4)^25^.

### TUNEL assay

Apoptotic cells in the cerebral I/R injury region were detected using a TUNEL assay. Briefly, whole brain tissue was harvested at 24 h after ischaemia and immediately fixed using 4% paraformaldehyde (n = 4/group). The fixed brain tissues were then sectioned coronally (thickness, 3 μm) and mounted on poly-l-lysine-coated glass slides. A commercially available TUNEL kit (Millipore, Billerica, MA, USA) was used according to the manufacturer's protocol for the detection of in situ apoptosis. Deparaffinised tissue sections were treated with proteinase K (20 μg/ml) for 15 min at room temperature; thereafter, endogenous peroxidase activity was quenched. The tissues were treated with a working solution containing a mixture of reaction buffer and terminal deoxynucleotidyl transferase (7:3) for 1 h at 37 °C, and then, the anti-digoxigenin peroxidase conjugate was treated at room temperature for 30 min. Subsequently, the tissues were treated with 3, 3′ diaminobenzidine tetrahydrochloride (Sigma-Aldrich) substrate (1:50) in the dark for 5 min and then stained with hematoxylin (Sigma-Aldrich) for 2 min in the dark.

The TUNEL + cells and total cells were observed under a microscope at a magnification of × 200 (ZEISS, HAL100, Germany) and counted using ImageJ (Bethesda, MD, USA) in three randomly selected fields from the IC and PIR samples. The percentage of TUNEL + cells was assessed using the ratio of TUNEL + cells to total cells.

### Immunofluorescence

To detect AhR and NeuN + cells, tissue sections were prepared using the same method as that for TUNEL staining (n = 4/group). Deparaffinised tissue sections were treated with a blocking solution (3% bovine serum albumin [Sigma-Aldrich] with 10% normal goat serum [Jackson ImmunoResearch, PA, USA]) for 1 h at room temperature. AhR immunodetection in brain tissues was performed using rabbit polyclonal anti-AhR (1:1,000; BML-SA210; Enzo Life Sciences, USA), and mouse monoclonal anti-NeuN antigen (1:200; MAB377; Merck, Germany) was used to detect NeuN + cells. The secondary antibodies used were goat anti-mouse IgG 594 (1:1,000; A32742; Thermo Fisher Scientific, USA) and goat anti-rabbit IgG 488 (1:1,000; A11008; Thermo Fisher Scientific). For DNA staining we used 4′,6-diamidino-2-phenylindole (DAPI) (1:10,000; D21490; Invitrogen, USA).

Image acquisition was performed using a laser scanning confocal imaging system (Zeiss LSM780, Germany), and image analysis was conducted using the ZEN 2.6 software (Zeiss). The expression of AhR and NeuN, in addition to DAPI nuclear staining, in the PIR was observed under a microscope at a magnification of × 630 (Zeiss LSM780). To quantify the nuclear fluorescent intensity of neuronal cells, the DAPI signal was used to manually mark individual nuclei showing NeuN staining^10^. Twelve neuronal cells per tissue were randomly selected to measure fluorescence intensity. Furthermore, we assessed neuronal loss in the IC and PIR. The number of NeuN + cells was observed under a microscope at a magnification of × 630 (Zeiss LSM780) and was counted in three randomly selected fields from the IC and PIR samples. All quantifications were analysed using the ImageJ software.

### Statistics

All data are expressed as means ± standard deviations (SDs). Statistical analyses were performed using the SPSS version 13.0 software (SPSS, Chicago, IL, USA). The infarct volume, rCBF, rADC, rT2, TUNEL + cells (%), AhR intensity, and NeuN + cells in multiple groups were compared using a one-way ANOVA with Tukey’s post-hoc test. Differences with a P value < 0.05 were considered statistically significant.

### Ethics statements

This study was performed following approval by the Institutional Animal Care and Use Committee of Asan Medical Center (IACUC Number: 2018-14-142). All experimental procedures were performed in strict accordance with the guidelines of the Association for Assessment and Accreditation of Laboratory Animal Care. All experiments are reported in compliance with the ARRIVE guidelines (Animal Research: Reporting in Vivo Experiments) for how to REPORT animal experiments. All surgeries were performed under isoflurane anaesthesia. All animals were closely monitored by trained individuals who were able to assess pain-related behaviours in animals. Euthanasia was planned if the animal exhibited persistent pain-related behaviour; however, no animals showed severe pain-related behaviour during the experimental period.

## Supplementary information

Supplementary information 1

Supplementary information 2

## Data Availability

The datasets generated during and/or analysed during the current study are available from the corresponding author by reasonable request.
