# Supplementary material for: Aryl hydrocarbon receptor antagonism before reperfusion attenuates cerebral ischaemia/reperfusion injury in rats
Source: Sci Rep. 2020 Sep 10;10:14906. doi: 10.1038/s41598-020-72023-5 (PMC7483549; doi:10.1038/s41598-020-72023-5)
Supplement: Supplementary file 2 — Supplementary information 2 [file 41598_2020_72023_MOESM2_ESM.docx]

**Aryl hydrocarbon receptor antagonism before reperfusion attenuates cerebral ischaemia/reperfusion injury in rats**

Jae-Im Kwon^1^, Hwon Heo^1^, Su Jeong Ham^1^, Yeon Ji Chae^1^, Do-Wan Lee^1^, Sang Tae Kim^2^, Joongkee Min^1^, Yu Sub Sung^3^, Kyung Won Kim^4^, Yoonseok Choi^5^, Dong Cheol Woo^2*^, Chul-Woong Woo^2*^

^1^Asan Institute for Life Sciences, Asan Medical Center, 88, Olympic-ro 43-gil, Songpa-gu, Seoul 05505, Republic of Korea

^2^Convergence Medicine Research Center, Asan Medical Center, 88, Olympic-ro 43-gil, Songpa-gu, Seoul 05505, Republic of Korea

^3^Clinical Research Center, Asan Medical Center, 88, Olympic-ro 43-gil, Songpa-gu, Seoul 05505, Republic of Korea

^4^Department of Radiology, Asan Medical Center, 88, Olympic-ro 43-gil, Songpa-gu, Seoul 05505, Republic of Korea

^5^Medical Research Institute, Gangneung Asan Hospital, 38, Bangdong-gil, Sacheon-myeon, Gangneung-si, Gangwon-do, Republic of Korea

*These authors contributed equally to this work.

**Corresponding authors**

Email: [dcwoo@amc.seoul.kr](mailto:dcwoo@amc.seoul.kr) (DCW)

Email: wandj79@hanmail.net (CWW)

Convergence Medicine Research Center

Asan Medical Center

88, Olympic-ro 43-gil, Songpa-gu, Seoul 05505, Republic of Korea

Phone: +82-2-3010-4155

Fax: +82-10-5559-7102

**Supplementary Table S1: Rates of tMCAO modelling success and reasons for exclusion of animals**

|  | **Control** | **TMF10** | **TMF50** |
| --- | --- | --- | --- |
| Success rate (%) | 61.5 (8/13) | 72.7 (8/11) | 80(8/10) |
| Causes for exclusion |  |  |  |
| 1. Death during operation | 1 | 1 | 0 |
| 2. Death after operation | 3 | 0 | 0 |
| 3. Insufficient rCBF reduction or no signs of infarction in MRI follow-up | 1 | 2 | 2 |

**Supplementary Table S2: rCBF changes at baseline and after the onset of ischaemia**

| Group | rCBF at baseline (perfusion unit) | rCBF after ischaemia perfusion (unit) | rCBF reduction (%) | p-value |
| --- | --- | --- | --- | --- |
| Sham | 167.1 ± 7.6 | 162.0 ± 10.2 | 3.1 ± 3.0 | - |
| Control | 168.5 ± 15.2 | 33.3 ± 8.6 | 80.1 ± 5.4 | 0.711 vs. TMF10 |
| TMF10 | 161.6 ± 12.8 | 28.8 ± 5.7 | 81.9 ± 4.7 | 0.862 vs. TMF50 |
| TMF50 | 167.9 ± 11.7 | 28.4 ± 5.1 | 83.1 ± 2.9 | 0.403 vs. Control |

**Supplementary TableS3: MRI sequence parameters**

|  | **T2-WI** | **T2 map** | **SE-EPI-DWI** |
| --- | --- | --- | --- |
| TR (ms) | 4000 | 3000 | 3000 |
| TE (ms) | 66 | - | 18.7 |
| Echo spacing (ms)/echo images | -/- | 10/15 | -/- |
| Averages | 2 | 1 | 2 |
| FOV (mm) | 25 x 25 | 25 x 25 | 25 x 25 |
| Acquired resolution (µm) | 260 | 260 | 260 |
| Rare factor | 16 | - | - |
| Slice number | 16 | 16 | 16 |
| Slice thickness (mm) | 1 | 1 | 1 |
| B-values (s/mm^3^) | - | - | 0 and 800 |
| Duration (min:sec) | 0:40 | 2:24 | 4:48 |
